# A Balanced Tissue Composition Reveals New Metabolic and Gene Expression Markers in Prostate Cancer

**DOI:** 10.1371/journal.pone.0153727

**Published:** 2016-04-21

**Authors:** May-Britt Tessem, Helena Bertilsson, Anders Angelsen, Tone F. Bathen, Finn Drabløs, Morten Beck Rye

**Affiliations:** 1 St. Olavs Hospital, Trondheim University Hospital, Trondheim, Norway; 2 MI Lab, Department of Circulation and Medical Imaging, Norwegian University of Science and Technology (NTNU), Trondheim, Norway; 3 Department of Cancer Research and Molecular Medicine, Norwegian University of Science and Technology (NTNU), Trondheim, Norway; 4 Department of Urology, St. Olavs Hospital, Trondheim University Hospital, Trondheim, Norway; Northern Institute for Cancer Research, UNITED KINGDOM

## Abstract

Molecular analysis of patient tissue samples is essential to characterize the *in vivo* variability in human cancers which are not accessible in cell-lines or animal models. This applies particularly to studies of tumor metabolism. The challenge is, however, the complex mixture of various tissue types within each sample, such as benign epithelium, stroma and cancer tissue, which can introduce systematic biases when cancers are compared to normal samples. In this study we apply a simple strategy to remove such biases using sample selections where the average content of stroma tissue is balanced between the sample groups. The strategy is applied to a prostate cancer patient cohort where data from MR spectroscopy and gene expression have been collected from and integrated on the exact same tissue samples. We reveal *in vivo* changes in cancer-relevant metabolic pathways which are otherwise hidden in the data due to tissue confounding. In particular, lowered levels of putrescine are connected to increased expression of *SRM*, reduced levels of citrate are attributed to upregulation of genes promoting fatty acid synthesis, and increased succinate levels coincide with reduced expression of *SUCLA2* and *SDHD*. In addition, the strategy also highlights important metabolic differences between the stroma, epithelium and prostate cancer. These results show that important *in vivo* metabolic features of cancer can be revealed from patient data only if the heterogeneous tissue composition is properly accounted for in the analysis.

## Introduction

Cancer is a heterogeneous disease where the understanding of underlying molecular mechanisms characteristic for each individual type could be important to provide efficient personalized and targeted therapy or choice of treatment. Human tissue samples generate a molecular snapshot of tumor states which is essential for characterization of *in vivo* cancer heterogeneity that is lost in animal models and cell-lines [1]. This applies to studies of tumor metabolism in particular [2]. A common but complicating factor when analyzing human tissue samples is the heterogeneous mixture of various cells and tissue types which can confound results from molecular analysis. This risk of confounding applies not only to prostate cancer (PCa) tissue, but to most cancers tissue types.

A simplistic model divides the human prostate tissue into three different components; benign epithelium, stroma and cancer tissue. Based on this model, a differential molecular analysis between cancer and normal samples should ideally emphasize differences between the benign epithelium and the PCa tissue. However, in such differential studies, normal samples are composed of two tissue types (benign epithelium and stroma) while PCa samples consist of three tissue types (benign epithelium, stroma and PCa), all in various proportions. This introduces a systematic sample bias of increased stroma content in the normal samples which confounds the molecular differences between epithelium and cancer. This problem is generally acknowledged [3,4]. However, thorough assessment of the composition of these three tissue types is not routinely accounted for during sample harvesting, preparation and analysis of PCa tissue samples. Additionally, the tumor environment introduces changes to the surrounding stroma tissue, termed stromogenic cancer [5,6], which represent a fourth tissue component in prostate cancer samples, but was not considered in this study.

Previously presented strategies to handle sample tissue heterogeneity have generally used computational models to adjust each sample for the influence of various tissue types in differential analysis [7,8]. Such computational strategies can either require prior information on tissue composition [9–11], pre-defined gene signatures [12,13], or be purely data driven [14,15]. Computational models usually handle tissue heterogeneity by making adjustment to the original expression values prior to differential analysis. This increase the risk of introducing a model bias, especially when the signal from each tissue component is not homogeneous. This is the case for tissue samples from many cancers, including PCa [16–19].

In this study we apply an alternative and simpler approach to account for the confounding effect of stroma when comparing PCa samples with normal sample histology for differential analysis. The strategy is to construct datasets of cancer and normal samples where the average content of stroma is balanced between the two datasets. The intention is to attenuate the differential signal due to various amounts of stroma in the analysis, and emphasize the true molecular differences between cancer and normal tissue. Unlike most computational methods used for tissue heterogeneity adjustments, the strategy does not perform corrections to the original molecular expression values. However, pathological characterization of tissue composition (benign epithelium, stroma and PCa) for all samples is required.

In this study, fresh frozen prostate tissue slices were collected after radical prostatectomy and each tissue core sample was histopathologically evaluated for tissue composition prior to analysis [20]. The tissue was then analyzed by HR-MAS (high resolution magic angle spinning) MRS (magnetic resonance spectroscopy) allowing 23 quantified metabolites, followed by gene expression measurements by microarray. HR-MAS is a non-destructive method, which permits gene expression analysis to be performed on the exact same tissue sample [21]. This integration of gene and metabolite data offers a unique opportunity to investigate central molecular pathways in PCa.

This study illustrates that variations in stromal tissue composition can be a systematic confounding factor in molecular analysis when PCa and normal tissue samples are compared. This bias can be removed by balancing the stromal composition between the sample sets. Only when such tissue biases are accounted for, integrated differential changes in genes and metabolites in central metabolic pathways can be simultaneously revealed.

## Methods

### Sample collection, microarray and HR-MAS analysis

Samples were obtained using a standardized harvesting procedure previously described by Bertilsson *et al*. [20]. This procedure includes cutting and handling of frozen tissue slices removed from the prostate gland during prostatectomy. Tissue sample cores were carefully selected from the slices based on clinical histopathology. Additionally, histopathological assessment of the amount of cancer, stroma and benign epithelium was carried out on each sample prior to the HR-MAS and microarray analyses (S1 File). Microarray and HR-MAS protocols and analysis are thoroughly described previously in Bertilsson *et al*. and Giskeødegård *et al*., respectively [21,22]. The *complete* dataset (95 PCa samples and 34 normal samples) contained microarray expression measurements for 16,381 genes, and HR MAS metabolite concentrations for 23 different metabolites measured on the exact same tissue samples. The use of human tissue material was approved by the Regional Committee for Medical and Health Research Ethics approval no 4-2007-1890. Consent forms were obtained from all patients. Other ethical aspects regarding the specific samples used in this study have been described in previous publications [20–22]. The microarray gene experiment is published in Array Express with accession E-MTAB-1041. Metabolite concentrations measured on each samples is given in S2 File.

### Controlling the influence of stroma tissue by sorting the original *complete* data into, *balanced*, *unbalanced* and *unstratified* datasets

**Fig 1:**
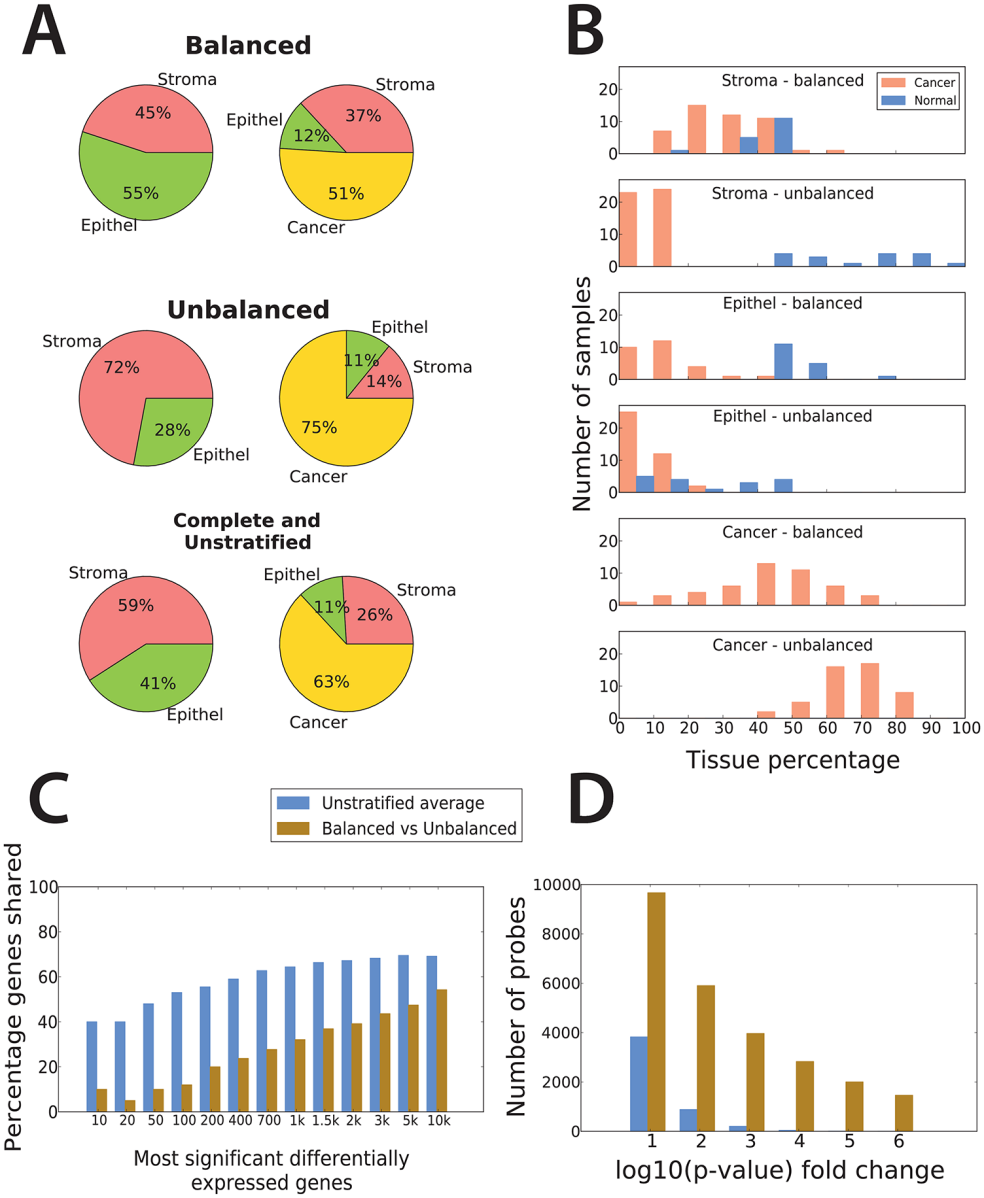
Average tissue composition in balanced and unbalanced datasets produce different sets of differentially expressed genes. (A) By canceling out the same types of tissues in PCa and normal samples, the *balanced* dataset compares in average 51% cancer to 43% benign epithelium and 8% stroma. Likewise, the *unbalanced* dataset compares 75% cancer to 58% stroma and 17% benign epithelium, while the *complete* and *unstratified* datasets compares 63% cancer to 33% stroma and 30% benign epithelium. The *balanced*, *unbalanced* and *unstratified* dataset have the same statistical power. Our strategy predicts that differentially expressed genes identified in the balanced dataset set should reflect changes in PCa rather than differences in tissue composition. (B) Histogram of tissue percentage distributions for cancer, stroma and benign epithelium in the *balanced* and *unbalanced* datasets. (C) Percentage of DEGs shared between the *balanced* and *unbalanced* datasets compared to the average percentage of DEGs shared between the 50 random subsets in the *unstratified* dataset. Percentages of shared genes are calculated using increasing numbers of the most significant DEGs in each dataset. The random numbers were calculated as the average number of genes shared over all 1225 possible comparisons between the 50 random subsets. For the 200 most significant DEGs, 20% were shared between the *balanced* and *unbalanced* datasets, and 39% were shared for the 2000 most significant DEGs. The corresponding percentages for the confounded dataset were 65% and 73% respectively. (D) Microarray probes with a p-value changing in various orders of magnitude between the *balanced* and *unbalanced* datasets greatly exceeds p-value changes expected by chance in the *unstratified* dataset. Of all probes, 2830 showed a p-value fold change of more than four orders of magnitude, and 1460 changed their p-values by more than six orders of magnitude between the *balanced* and *unbalanced* datasets compared to 42 and 1 probes for the *unstratified* dataset respectively.

The *balanced*, *unbalanced* and *unstratified* datasets were created by the following procedure: Cancer and normal samples were sorted independently according to their histopathologically determined content of stroma. The *balanced* dataset was created by splitting the sorted cancer and normal samples in two halves, and then selecting the 47 PCa samples with the highest stroma content (top half) and the 17 normal samples with the lowest stroma content (bottom half) from the sorted sample lists. This procedure minimizes the difference in average stroma content between PCa and normal samples for the *balanced* dataset (Fig 1A). Likewise, the *unbalanced* dataset was created by selecting the 48 PCa samples with the lowest stroma content and the 17 normal samples with the highest stroma content. In the *unbalanced* dataset, the difference in average stroma content between the cancer and normal samples is maximized (Fig 1A). The separation of samples into *balanced* and *unbalanced* datasets is given in S1 File. To create an *unstratified* dataset with the same statistical power as the *balanced* and *unbalanced* datasets, 50 random selections of 47 cancer and 17 normal samples were drawn from the *complete* dataset.

### Differentially expressed genes and metabolites

Raw expression values were filtered, log2 transformed and quantile normalized using the limma R package [23] as described in Bertilsson *et al*. [21]. All operations were performed prior to sample separations into *balanced*, *unbalanced* and *unstratified* datasets. Differentially expressed genes were identified for the *complete*, *unstratified*, *balanced* and *unbalanced* datasets as described in Bertilsson *et al*. [21]. P-values were corrected for multiple testing using Benjamini-Hochberg false discovery rate [24]. For the *unstratified* dataset, the average p-value over the 50 datasets was used. P-values for differentially expressed metabolites were calculated by the *t*.*test* function in R. The *t*.*test* function initially gives an assessment of whether the two sample groups display equal variance, which is used to decide the optimal significance test. Using the initial variance equality assessment, we used the *t*.*test* function with equal variance if the p-value of the initial test was below 0.05, and the test for unequal variance otherwise.

### Validation data

To validate the strategy of tissue balancing, an independent dataset [11] were downloaded from Gene Expression Omnibus (accession GEO ID, GSE8218). This data set consisted of microarray gene expression from 65 PCa samples and 71 normal samples together with histopathologic evaluation of four different tissue components (*prostate cancer*, *stroma*, *epithelium from BPH* and *atrophic gland*). To compare tissue composition in the validation set with the main dataset (the dataset used in the current study), percentages of *epithelium from BPH* and *atrophic gland* were combined into a single component corresponding to benign epithelium in the main dataset. Dataset balancing and identification of differentially expressed genes were performed in the same way as for the main data. To compare the similarity of differentially expressed genes highlighted in the *balanced* and *unbalanced* datasets between the validation and main data, we developed a *Significance Ratio (SR)* score calculated for each gene:

SR= pBpS (1)

for the *balanced* dataset and

SR= pSpB (2)

for the *unbalanced* dataset, where *p*_*B*_ and *p*_*S*_ are the p-value in the *balanced* and *unbalanced* datasets, respectively. The score is introduced to compare the relative p-values for each gene, and is calculated independently for the validation and the main data. Genes are ranked in ascending order based on the SR score for the *balanced* and *unbalanced* datasets in both the validation and main data. A high number of shared genes among the top-ranked genes in the main and validation data indicate that the compared lists have similar relative alterations in p-values between the *balanced* and *unbalanced* dataset.

## Results and Discussion

### Balancing the content of stroma tissue when human PCa tissue samples are compared to samples from normal prostate tissue

Samples from the original *complete* dataset are presented as three differently arranged subsets, *balanced*, *stroma* and *unstratified*, based on the difference in average stroma content between PCa and histopathologically normal samples (Fig 1A, Methods). The *balanced* dataset was designed to have an average stroma content as equal as possible between PCa and normal samples (37% vs 45% respectively). This was to emphasize the molecular transformations between cancer and normal tissue, without the confounding effect due to different amounts of stroma. In contrast, the *unbalanced* dataset was created with a maximized difference in average stroma content (14% vs 72% for PCa and normal samples respectively) to emphasize the molecular variations observed when the amount of stroma is not accounted for. A *unstratified* dataset was created with the same tissue properties as the *complete* dataset, but with a reduced number of samples to permit direct comparison of p-values with the *balanced* and *unbalanced* datasets. The *unstratified* dataset had an intermediate difference in average stroma content (26% vs 59% for PCa and normal samples respectively), representing the tissue composition in samples from a typical patient cohort. There was no significant difference in average Gleason score between the PCa samples in the *balanced*, *unbalanced* and *unstratified* datasets (7.17, 7.27 and 7.22 respectively). To simplify interpretation of the results, we assume that molecular signals from equal amounts of the same tissue type in PCa and normal samples cancel each other out in a differential analysis. This analysis does not take into account the changes between healthy stroma and stromogenic cancer. However, it will serve as a good approximation to study confounding signals from stroma due to different average tissue compositions in the cancer and normal samples. If we remove the contributions from equal amounts of tissue, analysis of the *complete* and *unstratified* datasets compare the molecular signal from 63% cancer tissue in the PCa samples to 33% stroma and 30% benign epithelium in the normal samples, which represents an almost complete confounding between stroma and benign epithelium. In this setting it is impossible to conclude whether the observed differentially expressed genes (DEGs) and metabolites are due to changes between PCa and normal tissue, or due to different amounts of stroma in PCa and normal sample groups. In contrast, the observed DEGs and metabolites are directly attributable to molecular changes between cancer and normal tissue for the *balanced* dataset, and cancer and stroma tissue in the *unbalanced* dataset. The differential signals in these two dataset comparisons thus highlight genes and metabolites, as well as the relationships between them, which are otherwise hidden in the *complete* and *unstratified* datasets.

Two criteria were used to divide samples between the *balanced* and *unbalanced* datasets: i) the average percentage of stroma should be as equal as possible between cancer and normal samples, and ii) each dataset should include as many samples as possible to ensure maximum statistical power. To improve the analysis even further, a third criterion can be introduced, which ensures that individual samples in each dataset are homogenous with respect to the percentage of stroma. This criterion should, in theory, reduce the expression value variance within each group to improve differential statistics. Due to the limited number of available samples with similar tissue composition, it was concluded that a selection also including the third criterion would compromise the statistical power of the analysis. Nevertheless, the first two criteria proved to be sufficient to highlight important differences between the *balanced* and *unbalanced* datasets.

An important feature of the *balanced* and the *unbalanced* datasets is that they indirectly also assess the difference between benign epithelium and stroma tissue. Genes and metabolites displaying differential change only in the *unbalanced* dataset, and not in the *balanced* dataset, are not markers for PCa transformation. These will rather be markers of stroma that changes in the *complete* and *unstratified* datasets only due to differences in the amount of stroma tissue. Further, genes and metabolites which display similar changes in both the *balanced* and the *unbalanced* dataset are markers for PCa which is unaffected by the presence of stroma. Finally, genes and metabolites displaying differential changes only in the *balanced* data set, but not in the *unbalanced* dataset, represent markers for PCa which are confounded by the presence of stroma. These assessments are important when separating observed changes directly related to PCa transformation from changes resulting only from biased amounts of stroma tissue. Such interpretations will be performed in the subsequent analysis.

### *Balanced* and *unbalanced* datasets display different patterns of gene expression at the global level

Considerable differences in gene expression patterns were observed when comparing DEGs between the *balanced* and *unbalanced* datasets. A far lower percentage of DEGs were shared between the *balanced* and the *unbalanced* datasets when compared to the percentage of DEGs expected to occur by chance (Fig 1B), where the expected percentage was estimated as the average number of shared genes between the 50 random subsets in the *unstratified* dataset. The fact that the *balanced* and *unbalanced* datasets capture significant biological differences which are unlikely to be observed by chance was confirmed by the high number of gene probes with a p-value changing by several orders of magnitude between the *balanced* and the *unbalanced* datasets compared to the *unstratified* dataset (Fig 1C). All three datasets displayed expected up and downregulation of seven well validated genes in PCa (Figure C in S3 File). To conclude, the observed differences between these datasets are attributable to differences in the amount of stroma tissue. The applied strategy of tissue balancing is able to highlight these differences.

### The *balanced* dataset highlights metabolites related to PCa change

**Fig 2:**
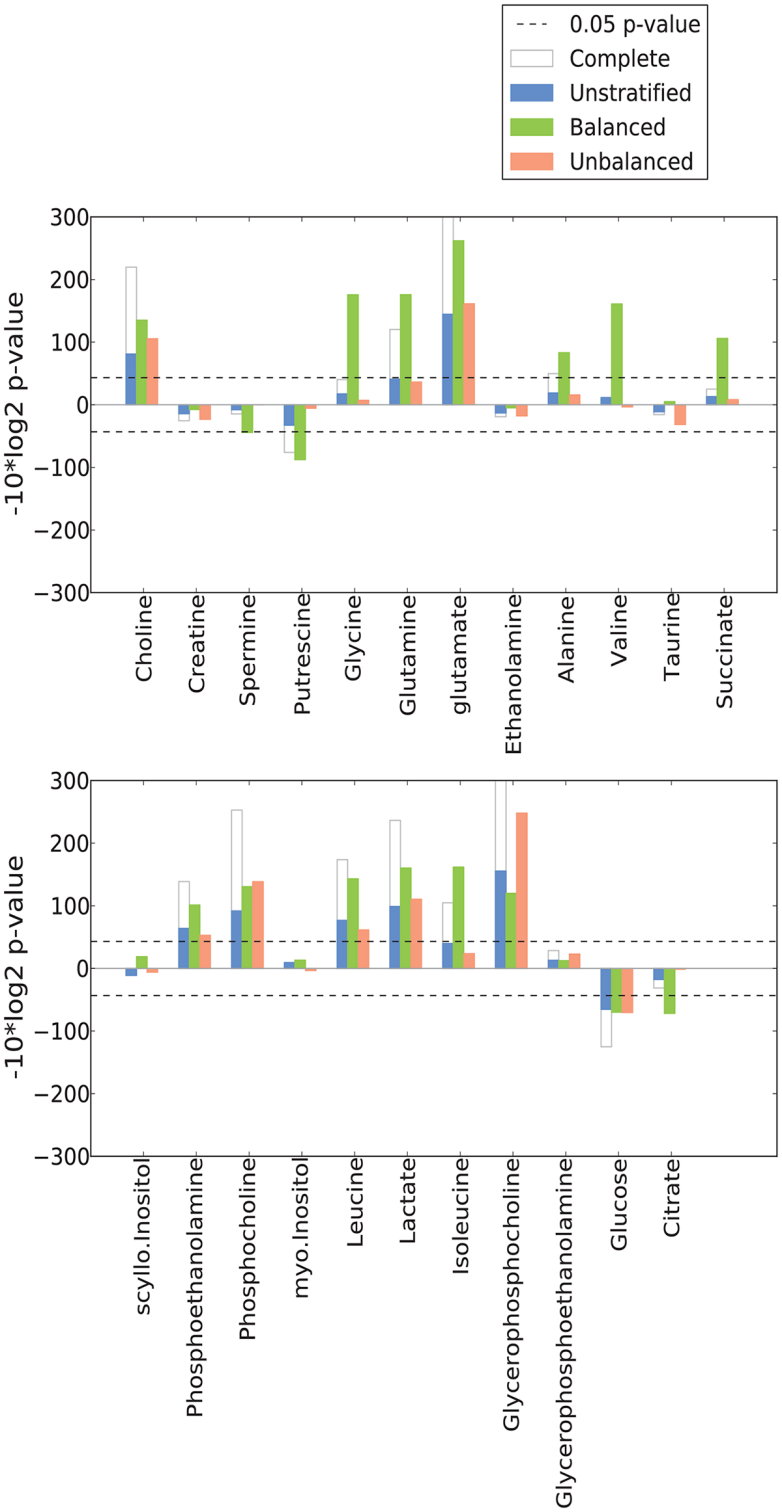
The *balanced* dataset improve the identification of significant metabolites altered in prostate cancer (PCa). Positive -log10(p-value) indicate increased concentration and negative -log10(p-value) indicate reduced concentration in PCa. The *unstratified*, *balanced* and *unbalanced* datasets all have the same statistical power.

In the *balanced* dataset 17 of the 23 metabolites measured by HR-MAS were found to display significant changes in concentration levels between PCa and normal tissue (Fig 2). This is a considerably higher number compared to the 8 significant metabolites identified in the *unstratified* and 13 identified in the *complete* dataset. This indicates a high degree of confounding from the stroma tissue in these datasets, which specifically applies to the nine metabolites putrescine, spermine, glycine, glutamine, alanine, valine, succinate, isoleucine and citrate that were significant in the *balanced* but not the *unstratified* dataset. These nine metabolites are thus changes which are characteristic for PCa but are difficult to detect due to confounding from unbalanced amounts of stroma tissue. The identification of citrate serves as a proof-of-principle for the applied analysis strategy, due to the absence of citrate in stroma and reduced levels observed in PCa tissue [22,25]. Additionally, changes in the metabolites succinate, valine and glycine were not detectable in the *complete* dataset, despite the higher statistical power compared to the *balanced* set. No metabolites were specific for the *unbalanced* dataset, possibly with an exception of taurine with borderline significance (p = 0.053). Other typical metabolite biomarkers for PCa like glucose, lactate and the three choline-containing metabolites [22,26] were observed in all datasets, thus showing stability as biomarkers regardless of tissue composition.

### Integration of metabolite and gene expression data pinpoints changes in genes involved in PCa metabolism

We predict that performing differential expression analysis in a dataset balanced for the content of stroma tissue between PCa and normal samples highlights genes and metabolites directly relevant for PCa changes. Moreover, the simultaneous gene and metabolite data measured on the exact same samples facilitate the integration of these data on specific metabolic pathways. In this study we focused on pathways of the TCA cycle, polyamine synthesis and fatty acid synthesis, and on DEGs related to the metabolites putrescine, citrate and succinate, as these pathways were specifically highlighted in the *balanced* dataset. Differential expression is presented in terms of p-values. Corresponding changes with respect to gene rank and fold change are presented in Figures A and B in S3 File and in S4 File.

#### Reduced putrescine levels relates to increased expression of *SRM* in the polyamine pathway

**Fig 3:**
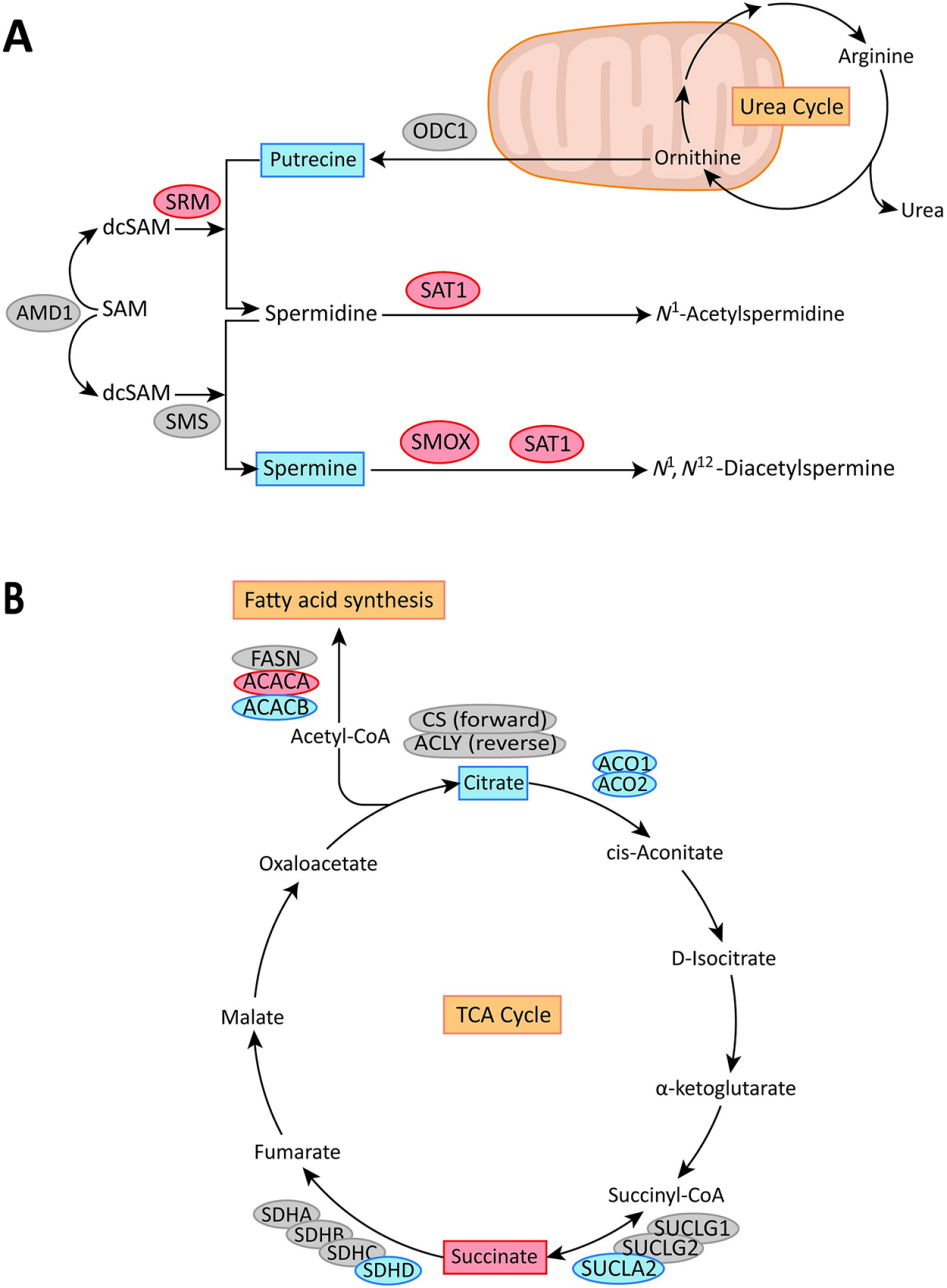
Schematic representation of pathways affected by genes and metabolites. (A) Polyamine pathway. (B) TCA cycle, fatty acid synthesis and glutamine consumption. The genes up- and downregulated in the *balanced* dataset are highlighted in red and blue respectively. Gene names: ODC1: ornithine decarboxylase 1, SRM: spermidine synthase, SMS: spermine synthase, AMD1: adenosylmethionine decarboxylase 1, SAT1: spermidine/spermine N1-acetyltransferase 1, SMOX: spermine oxidase, ACO1/2: aconitase 1/2, CS: citrate synthase, ACLY: ATP citrate lyase, ACACA/B: acetyl-CoA carboxylase alpha/beta, FASN: fatty acid synthase, SUCLA2: succinate-CoA ligase ADP-forming beta subunit, SUCLG1/2: succinate-CoA ligase beta subunit, SDHA/B/C/D: succinate dehydrogenase complex subunit A/B/C/D.

**Fig 4:**
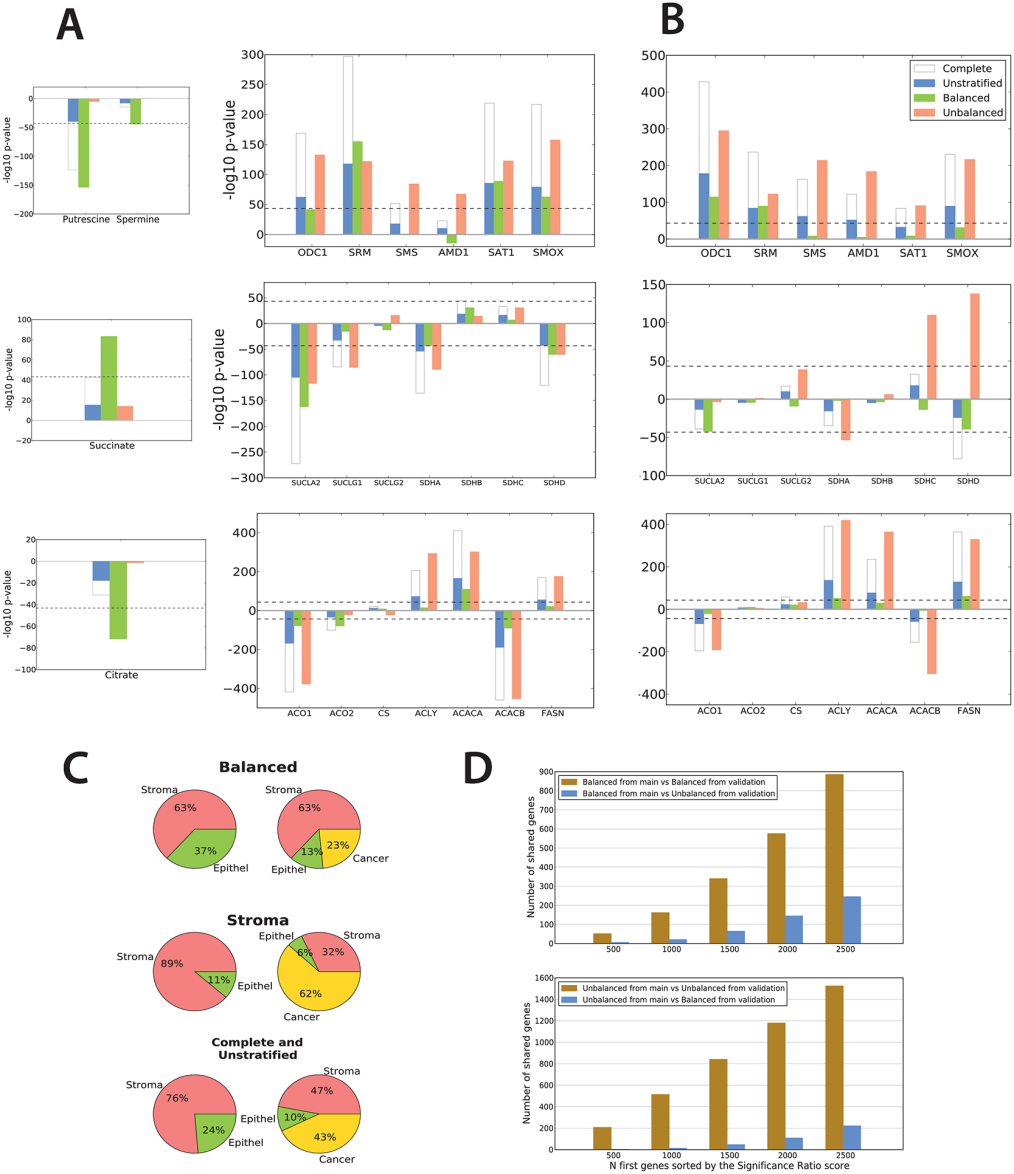
Integrated analysis of gene expression and metabolite concentrations highlights and validates genes corresponding to metabolic changes in key pathways important for prostate cancer (PCa). (A) Top: *SRM* and putrescine in the polyamine pathway. Middle: *SUCLA2* and *SDHD* and succinate in the TCA cycle. Bottom: Citrate in fatty acid synthesis. Positive -log10(p-value) indicate upregulation and negative -log10(p-value) indicate downregulation. The metabolites displayed on the left side are a subset of the metabolites shown in Fig 2. (B) Validation of significant genes in PCa compared to normal samples with respect to the polyamine pathway, succinate in the TCA cycle and fatty acid synthesis in an independent dataset. (C) Average tissue compositions in the *balanced*, *unbalanced* and *complete/unstratified* datasets from the validation study. Histogram of tissue distributions are shown in Figure D in S3 File. (D) Number of shared genes among the N first genes sorted by the significance ratio score when various datasets are compared. Genes with opposite changes (3 in the *balanced* and 116 in the *unbalanced* datasets) were removed from analysis. The number shared genes are much higher when *balanced* and *unbalanced* datasets are compared between themselves, than when *balanced* are compared with *unbalanced* datasets. This indicates that the validation and control studies display a highly concordant ranking of genes for both the *balanced* and *unbalanced* datasets.

Increased production of polyamines is important for cancer progression by promoting cell growth, degrading surrounding tissue and diminishing anti-tumor immune functions [27]. In the polyamine pathway (Fig 3A), *ODC1* converts ornithine to putrescine, which is further converted to spermidine by SRM. Spermidine is then converted to spermine by *SMS*, all in a forward reaction. *SRM* and *SMS* are assisted by *AMD1* in the conversion process, while *SAT1* and *SMOX* are responsible for the further downstream fate of spermidine and spermine [28]. Previous studies have reported that genes in the polyamine pathway are upregulated in cancer [29,30]. A concordant upregulation of polyamine genes will increase the flux of all polyamines, but not necessarily change the relative levels of individual metabolites. In the *balanced* dataset, we observe a significant level reduction for the metabolite putrescine. This reduction coincides with a specific upregulation of the *SRM* gene (Fig 4A). Mechanistically, upregulation of *SRM* will consume putrescine for the production of spermidine, and without putrescine replacement from ornitihine by upregulation of *ODC1*, the concentration of putrescine should be reduced. This is exactly what is observed from the MR metabolic measurements. Upregulation of SRM without concordant upregulation of other genes in the polyamine pathway is observed only in the *balanced* dataset, while a general upregulation of polyamine genes is a feature observed mostly in the *unbalanced* dataset. Applying the indirect relationship between the *balanced* and *unbalanced* dataset, this general upregulation are most likely caused by differences between benign epithelium and stroma, and the upregulation of SRM and concordant putrescine decline is due to PCa transformation. Interestingly, *SRM* has recently been suggested as a drug target for the polyamine pathway in a model of B-cell lymphomas [31]. In addition, a significant decrease in spermine levels (p = 0.047) is also observed, which fits with a significant upregulation of *SAT1* and *SMOX*, without significant upregulation of *SMS*. However, this relation is more subtle. A significant difference in spermine concentrations was previously reported to separate aggressive (Gleason grade ≥7) from more indolent types of PCa tissue (Gleason grade = 6) [22]. However, in this comparison the confounding of stroma is less pronounced since only cancer samples are compared.

#### Reduced citrate levels relates to increased fatty acid synthesis

Significantly higher levels of citrate in normal samples were only detectable in the *balanced* dataset. In the *complete* dataset, where the statistical power is doubled, citrate levels did not reach statistical significance between cancer and normal samples. Citrate is normally converted to isocitrate by *ACO1* and *ACO2* in the TCA cycle (Fig 3B), which is the preferred mode of energy production in most cells. The normal prostate deviates from this mode, because the conversion from citrate to isocitrate is partly blocked, leading to accumulation of citrate. Accumulated citrate can be secreted, or converted to acetyl-CoA and utilized for fatty acid synthesis (Fig 3B). Secretion leads to increased detectable levels of citrate, while higher TCA cycle activity and/or fatty acid synthesis reduces citrate levels. Our data supports a model where high level of citrate is a hallmark of normal prostate epithelial cells, and that citrate levels are reduced in both stroma and cancer cells.

The most pronounced gene differences related to citrate metabolism and fatty acid synthesis is found in the *unbalanced* dataset (Fig 4A). We therefore detect more gene differences between cancer and stroma than between cancer and benign epithelium. This result is in accordance with general TCA cycle activity in the healthy stroma [32], while TCA cycle function is altered in both benign epithelium and PCa. Concordantly, we observe a highly significant downregulation of *ACO1*, upregulation of fatty acid synthesis genes *ACLY*, *ACACA* and *FASN*, and downregulation of fatty acid oxidation gene *ACACB* in the *unbalanced* dataset where PCa are compared to the stroma. In the *balanced* dataset, changes in gene expression are more subtle. We observe significant downregulation of *ACO1*, *ACO2* and *ACACB*, and upregulation of fatty acid synthesis gene *ACACA*, but not *ACLY* and *FASN*. Altogether, this indicates that increased fatty acid synthesis is a hallmark feature for both the benign epithelium and PCa.

In the transformation to PCa (the *balanced* dataset) fatty acid synthesis can be further activated by increased expression of *ACLY*, *FASN and/or ACACA* (the rate limiting enzyme of fatty acid synthesis), leading to increased consumption of accumulated citrate. Consumption of citrate for fatty acid synthesis, rather than restoration of normal TCA cycle function may be the main reason for citrate depletion, since downregulation of both *ACO1* and *ACO2* in the *balanced* dataset corroborates decreased forward TCA cycle. This conclusion is in agreement with other studies highlighting increased fatty acid synthesis as a common feature in PCa [33]. However, compared to these studies, *ACACA* rather than *FASN* [33–35] is here highlighted as the main source of increased fatty acid synthesis. Inhibition of *ACACA* in PCa cell-lines induced growth arrest and apoptosis [36,37]. ACACA has also been shown to be a target for androgen receptor *AR* [38], and may be a drug target in PCa that directly targets *in vivo* changes in fatty acid synthesis.

#### Increased succinate levels relates to changed expression of *SUCLA2* and *SDHD*

Increased succinate levels were only observed in the *balanced* dataset. In the forward TCA cycle, the genes *SUCLG1*, *SUCLG2* and *SUCLA2* participate in the reversible conversion of succinyl-CoA to succinate, which is further converted to fumarate by the four succinate dehydrogenases *SDHA/B/C/D* (Fig 3B). Downregulation of these genes is most pronounced in the *unbalanced* dataset, especially for *SUCLA2*, *SUCLG1*, *SDHA* and *SDHD*. The cancer tissue is therefore more different from stroma than from benign epithelium, and this study indicates that stroma cells harbor canonical TCA cycle metabolism whereas benign epithelium and PCa do not. In benign epithelium and PCa these genes are downregulated, due to impaired TCA cycle and possibly increased fatty acid synthesis (Fig 4A). In the *balanced* dataset, downregulation of *SUCLA2* was particularly pronounced. Downregulation of *SUCLA2* without concordant downregulation of the related enzymes may disturb the metabolic flux balance, leading to the observed increased levels of succinate in PCa. However, since the conversion of succinyl-CoA to succinate is a reversible reaction, the actual effect of *SUCLA2* expression will depend on additional factors such as the equilibrium state between succinate and succinyl-CoA. A decreased, but less pronounced, expression of *SDHD* is also observed in the *balanced* dataset. In contrast to *SUCLA2*, conversion of succinate to fumarate by *SDHD* is irreversible, and downregulation of this enzyme will lead to accumulation of succinate in PCa. Earlier studies have suggested a tumor promoting function of increased succinate levels through activation of hypoxic regulatory programs by the transcription factor *HIF1A*, but did not mention PCa explicitly [39]. It is thus concluded that this mechanism, as well as other possible functions of increased succinate levels has yet to be explored for PCa.

### Validation in an independent cohort

Currently there exist only one additional dataset for PCa where the tissue composition of cancer, stroma and benign epithelium are characterized in both PCa and normal samples [11]. This enables a direct transfer of the balancing strategy applied in this study. Gene expression using microarrays were measured on 65 PCa and 71 normal tissue samples in this dataset, however no measurements of metabolism was collected and only validation of gene expression was possible. To our knowledge, there are no other study which simultaneously measure gene expression and metabolism in PCa. In both studies, histopathology showed the same bias of increased stroma tissue in the normal samples (Fig 4C). Using the 9723 genes with measurements in both datasets, concordant variations in differentially expressed genes (ranked according to the SR-score) were identified in the *balanced* and the *unbalanced* datasets (Fig 4D, Methods).

At the pathway level, we observed similar patterns for both the polyamine and fatty acid synthesis in the *balanced* and *unbalanced* datasets of both studies (Fig 4B). At the individual gene level, the specific cancer-related increase in expression of *SRM* in the polyamine pathway, as well as a general activation of all pathway genes in epithelium compared to stroma, was confirmed. In the fatty acid synthesis pathway, the validation analysis highlights *FASN* and *ACLY* as significantly upregulated, but not *ACACA* (p = 0.13). This supports the notion of increased fatty acid synthesis in PCa, but also indicates that this activity can be caused by the activation of various key genes in this pathway. Both datasets strongly confirmed increased fatty acid synthesis and downregulation of *ACO1* as a feature mostly characteristic of PCa when compared to stroma, while the transformation from epithelium to PCa involves more subtle changes. Succinate and the TCA-cycle showed less similarity and lower p-values for the involved genes. The expression of succinate genes also showed a comparable expression pattern in the validation data, though *SUCLA2* and *SDHD* were only borderline significant (p = 0.054 and p = 0.066 respectively).

Generally, the number of differentially expressed genes for the *balanced* dataset were fewer in the validation data compared to the main data (1011 versus 2800 respectively), and the p-values were less significant. This is likely attributed to the higher average content of normal stroma in the validation data (74% and 46% in validation and main data, respectively), which attenuates the differential signal. Overall, the validation datasets generally confirm the findings in the main data, and thus supports the viability of the proposed strategy to analyze tissue sample collection with large variations in tissue compositions.

An advantage of the applied strategy of removing stroma confounding the reduced amount of samples needed to obtain similar results of differentially expressed genes. To illustrate this, we performed differential expression analysis on the *complete* dataset using the histopathological analysis as contrasts in a factorial design (S4 File). When PCa were compared to benign epithelium, p-values in this analysis were highly comparable to the p-values in the *balanced* analysis. Since the influence of confounding tissue is under control in the *balanced* dataset, comparable p-values could be obtained by using only half the number of samples.

## Conclusion

A strategy to balance the original dataset based on pathology-reviewed tissue composition has been applied. Separation of changes between PCa and normal tissue, from changes between PCa and stroma, makes it possible to identify relevant DEGs and metabolites characteristic for each tissue component. A unique integration of gene expression and metabolite data on the exact same tissue sample highlights new information on important cancer pathways which can be possible future drug targets. The presented strategy is transferable to all heterogeneous cancer tissue types and similar results may be expected. The results from *balanced* and *unbalanced* datasets also show that adding more samples to a cohort study will not improve differential expression analysis unless the sample groups have a balanced composition of confounding tissue.

## Supporting Information

S1 File Sample metadata. Sample histopathology and assignment to *balanced* and *unbalanced* datasets.
(XLSX)

S2 File Metabolite concentrations. Metabolite concentrations measured by HR-MAS for each sample.
(XLSX)

S3 File Supplementary Figures A-D. Supplementary figures referenced in the manuscript, and figure captions.
(PDF)

S4 File Extended gene analysis table. Table of gene p-values, fold changes and gene ranks for the genes analyzed.
(XLSX)
