# Supplementary material for: A Balanced Tissue Composition Reveals New Metabolic and Gene Expression Markers in Prostate Cancer
Source: PLoS One. 2016 Apr 21;11(4):e0153727. doi: 10.1371/journal.pone.0153727 (PMC4839647; doi:10.1371/journal.pone.0153727)
Supplement: S3 File — Supplementary figures referenced in the manuscript, and figure captions. (PDF) [file pone.0153727.s003.pdf]

Supplementary File 3 – Supplementary Figure A-D

Captions

Figure A: Gene log2 expression values for cancer and normal samples in the *balanced* and *unbalanced* datasets for the genes in the polyamine, TCA cycle and fatty acid synthesis pathways analyzed in this study. Results are presented both for the main and validation data.

Figure B: Gene ranks for the *balanced* and *unbalanced* datasets for genes in the polyamine, TCA cycle and fatty acid synthesis pathways analyzed in this study. Results are presented both for the main and validation data.

Figure C: Validation of significant gene expression for genes commonly implicated in PCa in the *complete*, *unstratified*, *balanced* and *unbalanced* datasets. Gene names: AMACR: alpha-methylacyl-CoA racemase, KLK3: kallikrein related peptidase 3, GSTP1: glutathione S-transferase pi 1, MSMB: microseminoprotein beta, ERG: v-ets avian erythroblastosis virus E26 oncogene homolog, MYC: v-myc avian myelocytomatosis viral oncogene homolog, HPN: hepsin.

Figure D: Histogram of tissue percentage distribution for cancer, stroma and benign epithelium in the *balanced* and *unbalanced* datasets in the validation data.

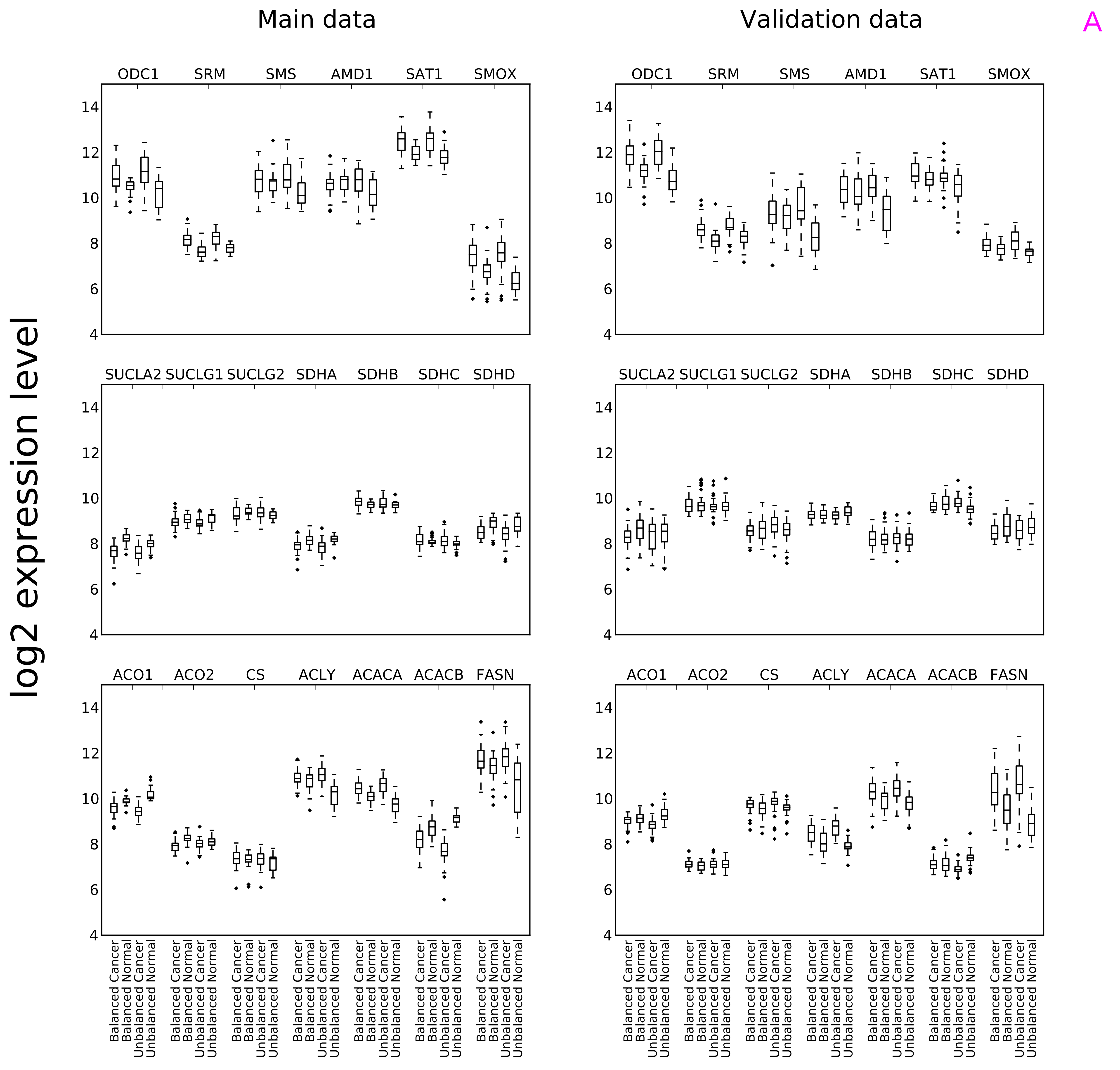

Main data
Validation data
A
log2 expression level
ODC1
SRM
SMS
AMD1
SAT1
SMOX
SUCLA2
SUCLG1
SUCLG2
SDHA
SDHB
SDHC
SDHD
ACO1
ACO2
CS
ACLY
ACACA
ACACB
FASN
Balanced Cancer
Balanced Normal
Unbalanced Cancer
Unbalanced Normal

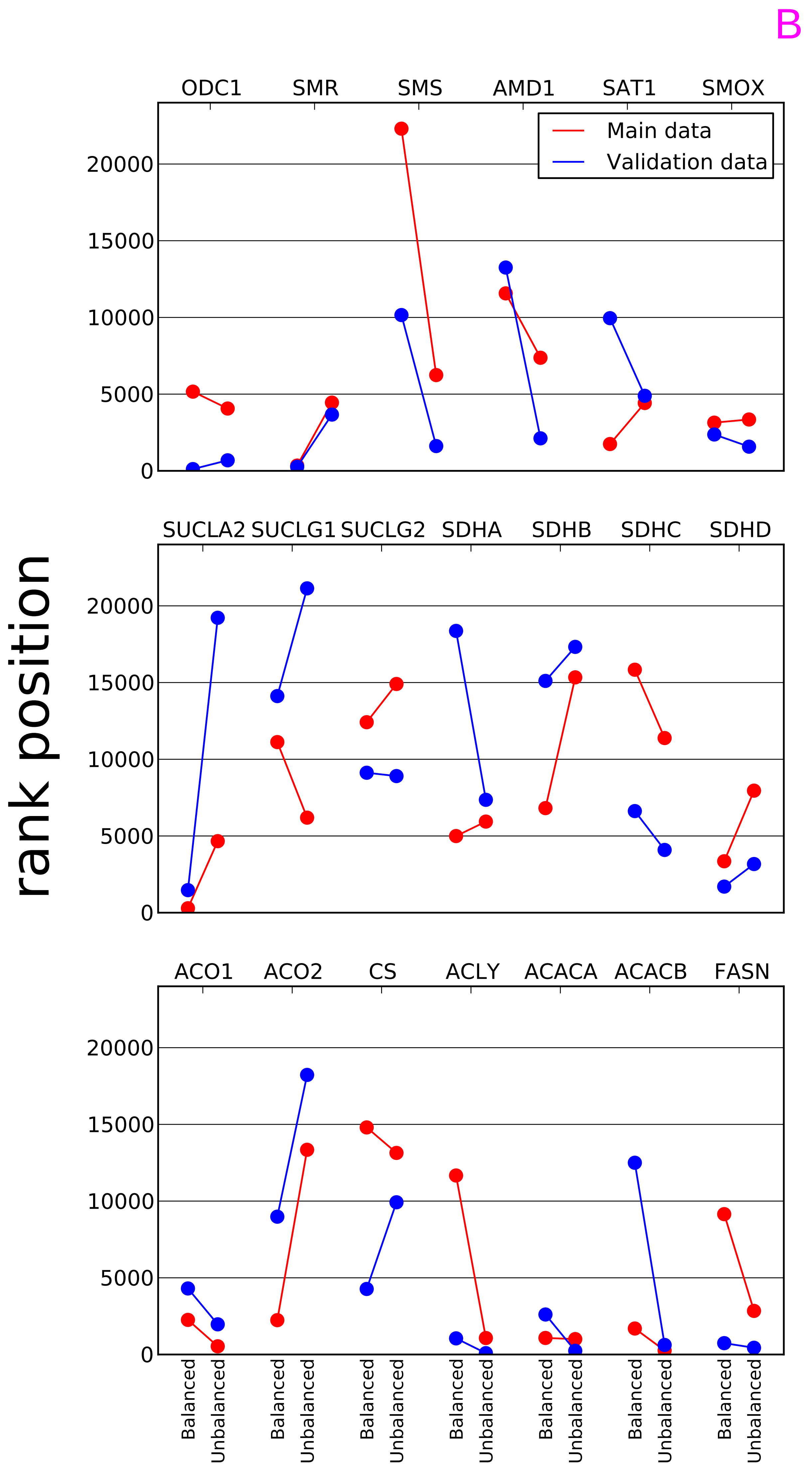
B
rank position
ODC1
SMR
SMS
AMD1
SAT1
SMOX
Main data
Validation data
SUCLA2
SUCLG1
SUCLG2
SDHA
SDHB
SDHC
SDHD
ACO1
ACO2
CS
ACLY
ACACA
ACACB
FASN
0
5000
10000
15000
20000
Balanced
Unbalanced

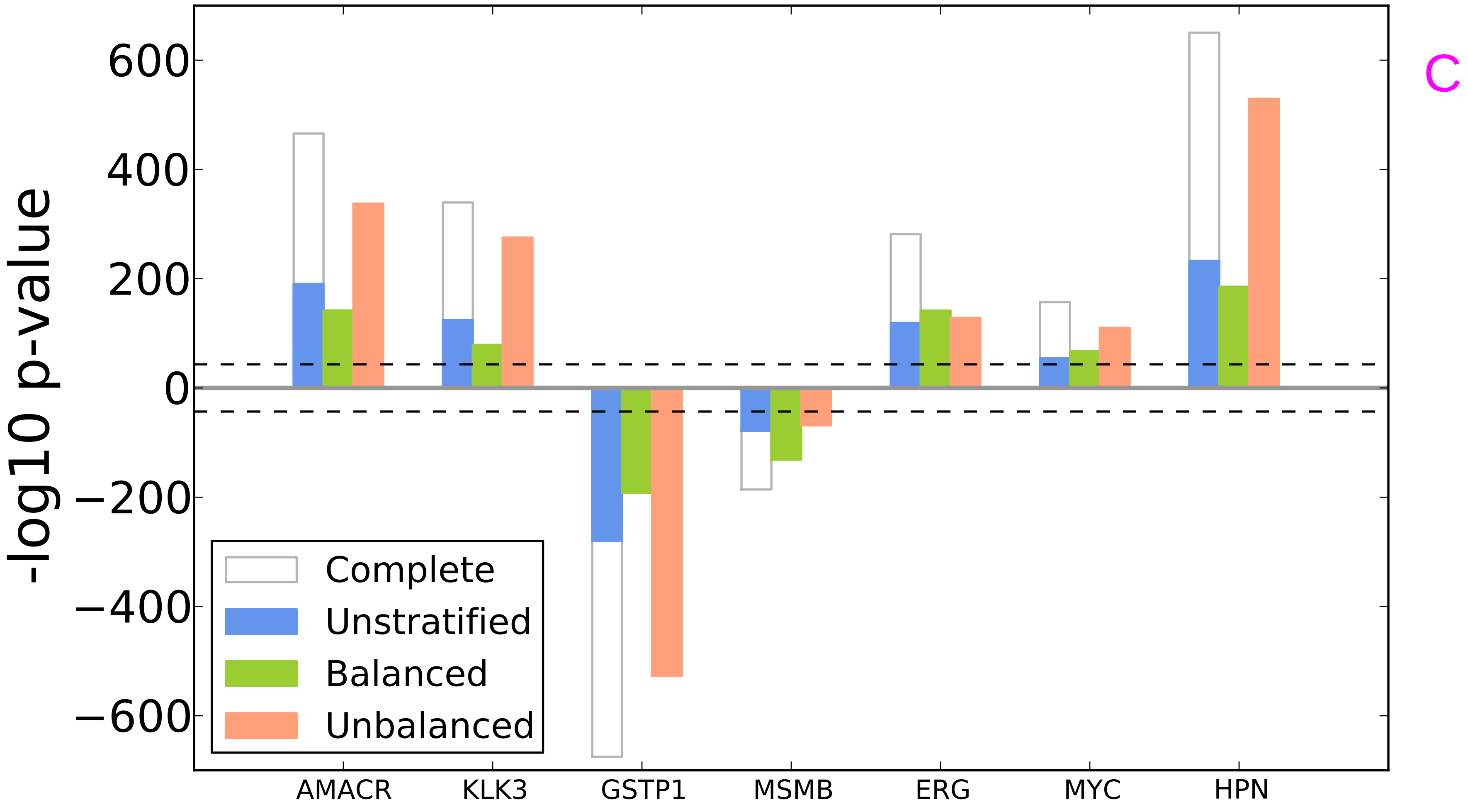

-log10 p-value
600
400
200
0
−200
−400
−600
AMACR
KLK3
GSTP1
MSMB
ERG
MYC
HPN
Complete
Unstratified
Balanced
Unbalanced
C

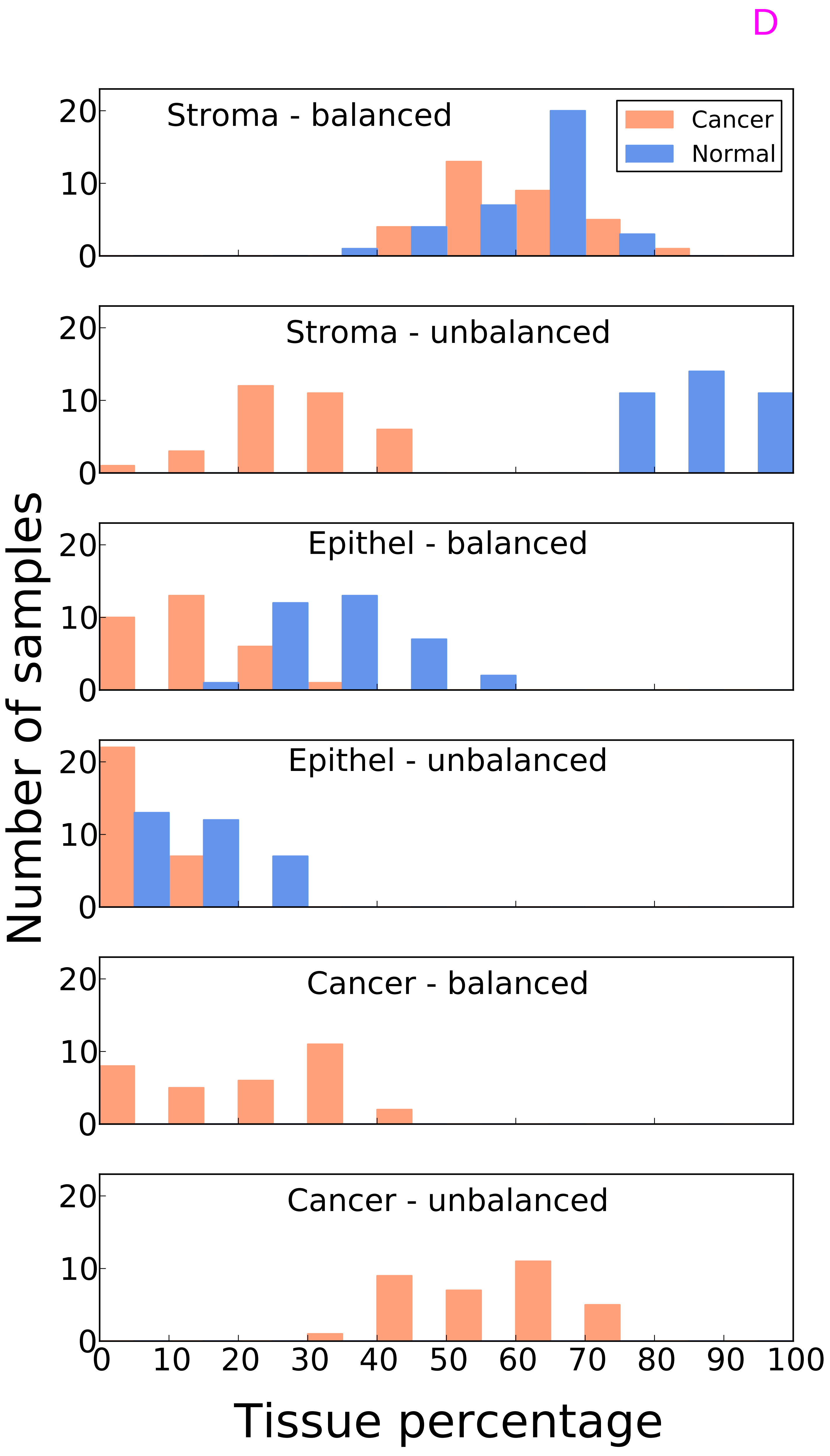
D
Stroma - balanced
Cancer
Normal
Stroma - unbalanced
Epithel - balanced
Epithel - unbalanced
Cancer - balanced
Cancer - unbalanced
Number of samples
Tissue percentage
0
10
20
30
40
50
60
70
80
90
100
